# Effect of various levels of date palm kernel on growth performance of broilers

**DOI:** 10.14202/vetworld.2017.227-232

**Published:** 2017-02-19

**Authors:** Muhammad Hamza Tareen, Rani Wagan, Farman Ali Siyal, Daryoush Babazadeh, Zohaib Ahmed Bhutto, Muhammad Asif Arain, Muhammad Saeed

**Affiliations:** 1 Department of Animal Nutrition, Faculty of Animal and Veterinary Sciences, Sindh Agriculture University, Tandojam, Pakistan; 2 Young Researchers and Elite Club, Tabriz Branch, Islamic Azad University, Tabriz, Iran; 3 Department of Animal Husbandary, Faculty of Veterinary and Animal Sciences, Lasbela University of Agriculture, Water and Marine Sciences, Uthal 3800, Pakistan; 4 Instititute of Animal Nutrition and Feed Technology, University of Agriculture, Faisalabad 38040, Pakistan

**Keywords:** broiler, date palm kernel, growth performance

## Abstract

**Aim:**

The aim of this study was the assessment of various levels of date palm kernel (DPK) on the growth performance of broilers.

**Materials and Methods:**

A 250-day-old broiler chicks were randomly selected and categorized into five groups (50 chicks/group) contained A (control), B, C, D and E fed with 0%, 1%, 2%, 3% and 4% levels of DPK in balanced ration, respectively, for 6 weeks. Feed and water intake were recorded daily in the morning and evening. The data for feed intake, water intake, live body weight, and feed conversion ratio (FCR) were recorded from all birds regularly. The carcass weight and percentage obtained via six slaughtered birds were randomly selected from each group. Finally, economic aspects of the rations evaluated.

**Results:**

The most feed intakes of broilers were recorded in Group A (3915.1 g) that was significantly higher than Groups D and E. The highest water intake was in Group E (9067.78 ml) that was significantly higher than Group A and control group. Live body weight was highest in Group E (979.85 g) than Groups B, C, and control group. The best growth weights were determined significantly in Groups D (1921.96 g) and E (1935.95 g). The lowest FCRs were indicated significantly in Groups D (1.97 g/g) and E (1.92 g/g) than Groups B and A. The highest carcass weights were recorded in Groups D (1214.01 g) and E (1230.88 g) that were significantly more than other groups. Dressing percentages in Groups D (61.76%) and E (62.17%) were higher than other groups (p<0.05). The net profits (Rs.) in Groups A, B, C, D and E were indicated 27.01, 32.77, 36.78, 43.47 and 44.51 per broiler, respectively.

**Conclusion:**

It was concluded that the high levels of DPK (3-4%) significantly decreased broiler feed intake and increased water intake, live body weight, growth weight, carcass weight, dressing percentage, net profit per bird and also had positive effects on growth of broilers.

## Introduction

Poultry Industry is the second largest industry in Pakistan [1], which has an important effect in bridging the gap between the supply and demand for protein in this country [2]. There many different locally available feed resources and raw material for poultry and domestic animals in Pakistan. The energy and protein rich cereal grains are studied for making the poultry feed [3]. The utilization of fruit byproducts and wastes gained significance, particularly use of these wastes as an ingredient in livestock feed [4,5]. Date palm is an important fruit crop produced abundantly in tropics and subtropics and it also grows in some areas of Pakistan. The previous use of date palm seed or kernel has not been reported in livestock feed particularly and poultry in Pakistan. The use of fruit waste products was helpful for declination of dependence on pricey raw materials used for feed composition, could manage with worldwide policies of depending on agro-industrial by-products for animal feed to reduce competition with man for cereals and to facilitate diminishing environmental pollution [6,7]. Date pits stones, kernels, or seeds of the date fruit which can reduce the cost of feed [6]. Date pits are cheap by-products with high energy content that can provide a potential alternative for conventional energy feed ingredients in the Poultry Industry. The nutritive chemical composition of date pits is as 8%, 7.5% fat, 5.8% protein content and contain 0.17% methionine, 0.31% lysine and 0.36% threonine compared to 0.15%, 0.27% and 0.31%, respectively, for maize [7-9]. A date kernel contains about 10-20% fiber, 10-20% crude protein (CP), and 55-70% nitrogen free extract (NFE) mainly starch, depending on date species [10-13]. The date palm kernel (DPK) is rich in protein, fat and dietary fiber; it contains 5.1, 9.0 and 73.1 mg/100 g DPK, respectively [14]. It can be added in the diet of livestock for augmenting the nutritional worth [15]. The high content of NFE in date kernels has attracted the attention of a number of researchers to evaluate its potential use in animal feed, with promising results. The previous studies reported in animals, chickens and quail suggested that date palm fruits, dates meal and meat, and waste products supplemented in the diet produced an effect on growth performance [16-19]. It can be noticed from the studies carried out by various researchers, that DPK or seed was considerably valuable to improve the growth and production performance, feed cost and feed utilization in poultry, when added in the feed on the basis of feed formulation [6,20]. In Pakistan, the date palm fruit is abundantly produced in few areas, but DPK is wasted and not included into the animal and poultry feed. This prompted us to assess the effect of different levels of DPK on growth performance of broilers.

## Materials and Methods

### Ethical approval

The present research was conducted after approval of Institutional Animal Ethics Committee of Animal Nutrition Laboratory, Sindh Agriculture University, Tandojam, Pakistan.

### Animals

The 250-day-old broiler chicks (Ross 308) were randomly selected and categorized into five groups (50 chicks/group) contained A (control), B, C, D and E fed with levels of 0%, 1%, 2%, 3% and 4% of DPK in the ration, respectively, for 6 weeks.

### Experimental design

Before conduction of the experiment, DPK was purchased from a local market and crushed and finally brought to the Animal Nutrition Laboratory, Sindh Agriculture University, Tandojam, Pakistan. The determination of CP, crude fiber (CF), and NFE on the dry matter (DM) basis was carried out according to the previously described method [21]. The crushed DPK contained 6.71% CP, 16.19% CF, and 59.70% NFE. After chemical composition, the rations were mixed with 1%, 2%, 3%, and 4% of DPK (Tables-1 and 2). The 250-day-old broiler chicks were divided into five Groups A, B, C, D and E (50 chicks/group). The Group A served as control and received the basal diet without DPK, while the other groups (B, C, D and E) received diets that supplemented with different levels of DPK feed. All the chicks were managed into semi-intensive housing system for 6 weeks provided with 1 square foot space per bird. During the 1^st^ week, chicks were grown at 95°F temperature later reduced by 5°F each week, finally maintained at 70°F as house temperature. The wooden dust litter was used for bedding. On day first flushing was practiced. Ad libitum feed and water was provided to all the chicks and light was provided for 24 h. Feed and water intake were recorded daily in the morning and evening for each group. The chicks were vaccinated according to approved schedule. At last day of the study, the data for feed conversion ratio (FCR) and live body weight were calculated and recorded, respectively, for all birds. The carcass weight and dressing percentage obtained at the end of study in six slaughtered birds that were randomly selected from each group.

**Table-1:** Experimental starter ration (%) of broiler chicken included various levels of DPK.

Ingredients	Groups					

	A (control)	B (1% DPK)	C (2% DPK)	D (3% DPK)	E (4% DPK)
Rice	25	25	22	23	23
Maize	24.5	23	26.2	25.7	25
Rice polish	5	5.5	5	5	5
Soya bean meal	7	7	6	7	5
Guar meal	5	5	5	5	4
Canola meal	13.5	13.5	13.5	11	11.5
Sun flower meal	6.5	6.5	6	5	5
Rape seed meal	3.5	3.5	3.5	3	3
Lime stone	1	1	0.8	0.7	0.5
DPK (crushed)	-	1	2	3	4
Fish meal	9	9	10	11.6	12
Corn gluetine (60%)	-	-	-	-	2
Total	100	100	100	100	100
Chemical composition of starter ration					
CP	21.25	21.18	21.1	21.05	21.2
Metabolizable energy	2851	2853	2852	2850	2854
Lysine	1.095	1.095	1.09	1.1	1.098
Methionine	0.442	0.44	0.44	0.45	0.47
Cystine/methionine	0.72	0.71	0.718	0.71	0.73
Calcium	1.06	1.06	1.04	1.088	1.03
Phosphorus	0.51	0.51	0.55	0.59	0.6

CP=Crude protein, DPK=Date palm kernel

**Table-2:** Experimental finisher ration (%) of broiler chicken included various levels of DPK.

Ingredients	Groups					

	A (control)	B (1% DPK)	C (2% DPK)	D (3% DPK)	E (4% DPK)
Rice	30.1	30.1	27.1	28.1	28.1
Maize	22.6	21.1	24.3	23.8	23.1
Rice polish	9	9.5	9	9	9
Soya bean meal	3	3	2	3	1
Guar meal	4	4	4	4	3
Canola meal	12.8	12.8	12.8	10.3	10.8
Sun flower meal	6	6	5.5	4.5	4.5
Rape seed meal	3	3	3	2.5	2.5
Lime stone	0.3	0.3	0.1	0.2	0.3
DPK (crushed)	-	1	2	3	4
Fish meal	8.7	8.7	7.7	11.3	11.7
Corn gluetine (60%)	0.5	0.5	2.5	0.3	2
Total	100	100	100	100	100
Chemical composition of finisher ration					
CP	19.02	19.09	18.97	18.99	18.95
Metabolizable energy	2910.7	2912.2	2915.2	2911.2	2912.7
Lysine	0.952	0.952	0.947	0.957	0.955
Methionine	0.406	0.404	0.404	0.414	0.434
Cystine/methionine	0.69	0.68	0.672	0.68	0.70
Calcium	0.91	0.91	0.89	0.938	0.88
Phosphorus	0.51	0.51	0.55	0.59	0.6

CP=Crude protein, DPK=Date palm kernel

### Statistical analysis

All the obtained data were subjected to analysis of variance using the general linear model procedure of SAS (2001) and least standard difference (LSD) set as p<0.05 [22].

## Results

### Feed intake

Feed intake of broilers (Table-3) in Group A (control) was higher (3915.11 g/b) than birds in other groups. The lowest feed intake (3794.22g/b) was recorded in broilers Group E, respectively. The LSD test suggested that differences in feed consumption was not significant (p>0.05). The feed intake of broilers differed more significantly (p<0.01) when broilers in Groups A and D (3.0% DPK) were compared with broilers of Group E (4.0% DPK).

**Table-3:** The growth performance of broilers as influenced by feeding various levels of DPK for 6 weeks.

Parameters	Groups					

	A (control)	B (1% DPK)	C (2% DPK)	D (3% DPK)	E (4% DPK)
Feed intake (g/b/week)	3915.1^a^	3835.66^ab^	3839.42^ab^	3874.82^b^	3794.22^c^
Water intake (ml/b/week)	8203.14^b^	8264.56^b^	8612.16^ab^	8905.08^a^	9067.78^a^
Live body weight (g/b/week)	1765.31^c^	1819.87^bc^	1877.65^b^	1965.46^a^	1979.85^a^
Growth weight (g/b/week)	1720.21	1777.77	1835.55	1921.96	1935.95
FCR (g/g)	2.22^b^	2.11^b^	2.04^ab^	1.97^a^	1.92^a^
Carcass weight (g/b)	1063.74^c^	1104.81^bc^	1147.93^b^	1214.01^a^	1230.88^a^
Dressing %	60.25^c^	60.70^b^	61.14^b^	61.76^a^	62.17^a^

^abc^Means within each row with no common superscript differ significantly (p<0.05). FCR=Feed conversion ratio, DPK=Date palm kernel

### Water intake

The water intake in broilers (Table-3) in Group E was highest (9067.78 ml/b), followed by the broilers in Groups D (8905.08 ml/b) and C (8612.16 ml/b), respectively. The lowest water intake was noted in Groups B and A. The results in relation to water intake were highly and statistically significant between groups (p<0.01) as well as for weeks (p<0.001). During comparison between the groups, the non-significant difference was observed for Group E (4.0% DPK) compared with other groups. However, the differences were highly significant (p<0.01) when water intake of broilers fed with high levels of DPK (3% and 4%) compared with birds fed low levels of DPK (1% and 2%) and control group.

### Live body and growth weight of broiler

The live body weight (Table-3) of broilers in Groups E and D were higher than Groups C and B; Group A (1765.31 g/b) was the lowest in live body weight. Similarly, the growth weight of broilers recorded as in Groups E and D were higher than Groups C, B and A which suggested that DPK is an effective ingredient for broiler feed that reduces the feed intake and increases the live body weight and growth weight. The results further suggested that 3-4% of DPK could be adequately used as an ingredient for the broiler feed to get higher live body weight and growth weight over commonly used as commercial broiler feed. It can be noted that live body weight increased (p<0.01) with increasing level of DPK.

### FCR

FCR (Table-3) of broilers in Group E (1.92 g/g) was more efficient that followed by the broilers in Groups D (1.97 g/g), C (2.04 g/g), and B (2.11 g/g). Relatively poor FCR (2.22 g/g) was calculated in broilers of control group. The data showed that increasing level of DPK improved FCR of broilers considerably, which was mainly associated with lower feed consumption to produce highest live body weight. However, the difference in improvement of the feed conversion efficiency was not so pronounced when the broilers were fed ration containing 4% DPK. The effect of weeks on the FCR was statistically non-significant (p>0.05). The LSD test suggested that increasing the level of DPK in broiler feed caused improving the FCR (p<0.01).

### Carcass weight

The carcass weight (Table-3) was remarkably higher in Group E (1230.88 g/b), followed by Groups D (1214.01 g/b), C (1147.93 g/b), and B (1104.81 g/b). The lowest carcass weight was recorded in broilers of control group (1063.74 g/b). The significant difference (p<0.05) was seen when Groups D and E were compared with lower DPK treated groups (1% and 2%) and control group.

### Dressing percentage

The dressing percentage (Table-3) was relatively higher (62.17%) in Group E broilers, while Groups D, C and B indicated an average of 61.76%, 61.14% and 60.70%, respectively. The lowest dressing percentage was calculated in control group. The significant difference (p<0.05) in dressing percentage was noticed when Groups D and E were compared with Groups A and B.

### Economics

After completion of the experimental period of 42-day, all the broilers were marketed to know the economic viability of the ration containing various levels of DPK. The results (Table-4) showed that the total cost of production of broiler which included expenditures spent on purchasing day-old chicks, feed cost, cost on purchase of date DPK and other miscellaneous expenditures on broilers while the gross revenue earned from the marketing of these broilers in Groups A, B, C, D and E showing net profit of Rs. 27.01, 32.77, 36.78, 43.47 and 44.51/b, respectively. It was noted that maximum net profit (Rs. 44.51/b) was achieved from the broilers fed 4.0% DPK, closely followed by those broilers fed 3.0% DPK (Rs. 43.47/b).

**Table-4:** Economic aspects of broiler rations as affected by feeding various levels of DPK.

Particulars	Groups					

	A (control)	B (1% DPK)	C (2% DPK)	D (3% DPK)	E (4% DPK)
Cost of day old chick (Rs./b)	69.00	69.00	69.00	69.00	69.00
Total cost of feed (Rs./b)	101.79	99.73	99.82	100.75	98.65
Cost of crushed DPK	0.00	2.88	5.76	8.64	11.52
Miscellaneous expenditure (Rs/b)	14.00	14.00	14.00	14.00	14.00
Total production cost (Rs./b)	184.79	185.61	188.58	192.39	193.17
Final live body weight (Rs./b)	1.765	1.820	1.878	1.965	1.980
Broiler sale rate (Rs./kg)	120.00	120.00	120.00	120.00	120.00
Total income (Rs./b)	211.84	218.38	225.36	235.86	237.68
Net profit (Rs./b)	27.01	32.77	36.78	43.47	44.51

DPK=Date palm kernel

## Discussion

The date palm, mango, banana, orange, and tomato are the main fruits in tropical and subtropical counting Pakistan, and their by-products can be supplemented in livestock feeding in dry form [4]. The nutritional value of these fruits is predominantly essential, and therefore, can be formulated and balanced in ration of poultry [6,20]. The present research was conducted to establish the effect of various percentage of DPK on growth performance of broilers. One of the possible ways that might be caused a better performance in broilers is prevention of pathogens and generation of good microbes in *gastrointestinal* system [23]. Currently, in a study increasing palm kernel meal to 30% resulted in increased *Lactobacillus* spp. and reduced coliform/*Escherichia coli* counts (p≤0.005), and lower (p≤0.05) excreta pH [24]. This effect can cause some beneficial effects on performance of digestive system in birds and might be attributed to prevention of pathogens from colonizing in digestive tract via competitive exclusion [23,25].

The broilers treated with 3-4% DPK consequence high live body weight and FCR in contrast to broilers treated with 1-2% DPK. 1-4% of DPK in diet did not affect on health of broilers. Although several researchers [18,26,27] had reported that palm kernel meal (PKM) can be used up to 40% in poultry diets, in another study, it is concluded that just up to 7.5% of PKM level can be used in broiler diets for better economical production [28]. DPK treated groups consumed less feed and achieved more live body weight. Similarly, it is reported that adding 8% and 16% of palm kernel meal improved growth performance in broiler chicken and lowered FCR [11]. It might be chicks efficiently digest the feed and absorb the nutrients inside the gut. In a study indicated, inclusion of palm kernel meal into the basal diet, irrespective of inclusion level, enhanced starch and fat digestibility [20]. Regardless of current findings, it is reported that laying hens which included 5% and 10% palm kernel in their diet reduced the daily body weight gain but did influence affect on egg production [6]. Probably more proficient gut and physiological conditions of chicks’ body increased live body weight resulting in increased carcass weight and dressing percentage. However, the various levels of DPK can be more beneficial when supplemented in high rates in growing ration of broilers rather than in starter ration, because the digestive system is mature and completely functional. The results of this study coincide with the previous reports [16-18,29] which indicated the supplementation of DPK can cause a better performance at the end of rearing period.

Water intake is effective on broiler health, performance, and welfare status of birds [30]. Water availability is very essential to achieve efficient broiler performance rate. There are various factors that can affect on water intake in broilers, such as intake of minerals and feed [31], water quality and temperature [32]. The chicks in DPK treated groups consumed in significantly more water possibly due to the more efficient gut, on the other hand, DPK may cause more thirsty effect. It is observed that increasing water consumption can increase feed passage rate, and therefore, it may decrease nutrient absorption [33].

The cost of feed is not permanent and it can change by economic conditions of markets however the groups treated with 3-4% DPK in ration were more profitable which was in agree with other study that added 5% palm kernel to diet of laying hens and reduced the cost of feed effectively [6]. Regardless of the present findings in a survey indicated that feed cost per kilogram bird live weight was lowest for corn-soy-based ration compared to rations with various types of palm kernel in Malaysia [34]. In another study indicated that diets supplemented with 9% and 12% palm oil is very effective on weight gain and feed efficiency but concluded that this level of supplementation was too expensive and impractical for commercial use.

## Conclusion

It was concluded from this study that increasing the level of DPK in broilers rations caused positive effects on growth performance and reduced the feed intake. 3-4% of DPK was more safely in broilers production and also using palm kernel could reduce the cost of feed. However, the results of this study support and inferred the positive findings and conclusions about using palm kernel in broiler diet, but future studies can compare the palm kernel and palm oil with other feeding sources in broiler, breeder, and layer rations.

## Authors’ Contributions

RW designed the study and prepared the field condition. MS contributed in the data analysis. DB drafted and revised the manuscript. FAS and ZAB performed the fieldwork and collected the data. MHT and MAA gave valuable advices and support in the conduct of the study. All authors have read and approved the final manuscript.
